# Masking healthcare workers (HCW) and visitors was effective to reduce nosocomial (NOSO) influenza (I) during 2014/15 epidemic with reduced vaccine effectiveness

**DOI:** 10.1186/2047-2994-4-S1-O61

**Published:** 2015-06-16

**Authors:** A Iten, C Bonfillon, S Boymond, Y Thomas, S Cordey, C-A Siegrist, L Kaiser, D Pittet

**Affiliations:** 1 Infection Control Program, University of Geneva Hospitals, Geneva, Switzerland; 2 Hospital Health Service, University of Geneva Hospitals, Geneva, Switzerland; 3 Laboratory of Virology, University of Geneva Hospitals, Geneva, Switzerland; 4 Department of Child and Adolescent Medicine, University of Geneva Hospitals, Geneva, Switzerland

## Introduction

Vaccination of HCW against seasonal influenza (SI) is the cornerstone for the prevention of NOSO I. In countries where vaccination cannot be made mandatory by law, an alternative exists: HCWs’ obligation to be vaccinated (VAC) or to wear a mask during SI epidemics. This is the strategy (called “Zoning”) adopted by HUG since 2009. In Switzerland, in winters 2013/14 & 2014/15, SI had similar epidemic curves but vaccine effectiveness differed. In 2014/15, the trivalent vaccine did not cover the major circulating SI H3N2 strain.

## Objectives

We describe 2013/14 and 2014/15 SI epidemics and NOSO at HUG.

## Methods

Suspected cases of SI (respiratory symptoms, fever with chills, muscular pain, or prostration) were screened using nasopharyngeal samples analyzed by RT-PCR. Cases were defined as NOSO when symptoms occurred >72 h after admission. Regular audits were performed to assess compliance with recommendations.

## Results

In winter 2013/14, 309 patients were positive for I, 147 of which (47.6%) were NOSO. Droplet precautions with single room isolation whenever possible were implemented for 261 patients (84.5%). Of 4459 HCW observed, 78.5% were VAC or wore a mask. In winter 2014/15, “Zoning” was implemented on 31/12/2014. Early Jan 2015, a large number of SI was documented with a high proportion of NOSO: 49.2% (92/187), in particular in internal medicine (19/28; 67.8%). At time of audit, 992/1262 (78.6%) HCW were VAC or wore a mask. Additional measures were implemented from 15^th^ January to 20^th^ March 2015: mandatory mask for HCW (even for VAC HCW) and visitors. Following this additional measure, 68/175 (38.8%) cases were NOSO at HUG, in particular 19/121 (15.7%) in internal medicine. Recommendations were followed by 2143/2769 (77.4%) HCWs and 430/685 (62.8%) visitors. Droplet precautions were implemented for 432/468 (92.3%) SI patients.

## Conclusion

During the large 2014/15 epidemic with reduced SI vaccine effectiveness, mandatory mask wear for HCW and visitors was an effective measure to reduce NOSO I.

## Disclosure of interest

None declared.

